# The Impact of Anonymity in Emergency Medicine Morbidity and Mortality Conferences: Findings from a National Survey of Resident Physicians

**DOI:** 10.5811/westjem.2019.10.44497

**Published:** 2019-12-19

**Authors:** Emily L. Aaronson, Kathleen Wittels, Richard Dwyer, Eric Nadel, Fiona Gallahue, Olesya Baker, Christopher Fee, Robert Tubbs, Jeremiah Schuur

**Affiliations:** * Harvard Medical School, Massachusetts General Hospital, Department of Emergency Medicine, Boston, Massachusetts; † Harvard Medical School, Brigham and Women’s Hospital, Department of Emergency Medicine, Boston, Massachusetts; ‡ Lawrence Center for Quality and Safety, Massachusetts General Hospital and Massachusetts General Physicians’ Organization, Boston, Massachusetts; § University of Washington School of Medicine, Department of Emergency Medicine, Seattle, Washington; ¶ Center for Clinical Investigation, Brigham and Women’s Hospital, Boston, Massachusetts; || University of California San Francisco School of Medicine, Department of Emergency Medicine, San Francisco, California; # Warren Alpert Medical School at Brown University, Department of Emergency Medicine, Providence, Rhode Island

## Abstract

**Introduction:**

Although the Accreditation Council for Graduate Medical Education mandates structured case review and discussion as a part of residency training, there remains little guidance on how best to structure these conferences to cultivate a culture of safety, promote learning, and ensure that system-based improvements can be made. We hypothesized that anonymous case discussion was associated with a more effective, and less punitive, morbidity and mortality (M&M) conference. Secondarily, we were interested in determining whether this core structural element was correlated with the culture of safety at an institution.

**Methods:**

We conducted a national survey at 33 emergency medicine residency programs evaluating residents’ perceptions of M&M and the culture of safety at their institutions. Data was analyzed using descriptive statistics and bivariate analyses. We summarized Likert scores using mean and 95% confidence intervals. We also performed content analysis of the free-text comments and report on the themes identified.

**Results:**

There were 1248 residents at the 33 programs surveyed. Of the 1002 who replied (80.3% response rate), 231 respondents reported anonymous case presentations and 744 reported non-anonymous case presentations. Residents at programs with anonymous case presentations were more likely to report that M&M was non-punitive. There were no other significant differences between anonymous and non-anonymous case presentations on any of the culture of safety domains measured. When these comments were systematically analyzed and coded, we found that the comments related to anonymity were both positive and negative. Among the themes identified were anonymity’s impact on punitive response to error, the ability to learn from cases, and professional responsibility.

**Conclusion:**

Anonymous M&Ms are associated with a perception of a less-punitive M&M and with better ratings in several conference-specific outcomes; however, there appears to be no association between the other Agency for Healthcare Research and Quality culture of safety scores and anonymity in M&M.

## INTRODUCTION

The value of systematic error analysis has long been recognized in healthcare. Pioneered by Earnest Codman at the turn of the 20^th^ century,^1^ and famously reinforced 100 years later in the Institute of Medicine’s landmark *To Err is Human*,^2^ the importance of routine case reporting and detailed case review is now widely accepted as foundational in the practice of medicine. Integral in training, the Accreditation Council for Graduate Medical Education now mandates structured case review and discussion, or morbidity and mortality (M&M) conferences, as a part of their system-based practice and practice-based learning and improvement domains.^3^

Despite the express interest in this activity supporting system-based practice, M&Ms have instead traditionally been focused on individual cognitive errors and have often further reinforced a “blame and shame” culture in medicine, undermining the effectiveness of these conferences.^4^,^5^,^6^ For trainees in particular, it has been noted that the impact of M&M conferences that focus on individual cognitive errors is to increase fear of blame resulting in decreased participant engagement, lower likelihood of reporting safety events, and an overall decrease in the effectiveness of the conferences.^7^ Despite this, there are no best practices to guide residency programs in the design of M&M conferences to mitigate the fear of blame, and to promote a non-punitive case discussion. To our knowledge, no work to date has looked to determine the association of different structural elements of M&M conferences and residents’ perceptions of the conference and the overall impact on the culture of safety.

Recent papers have described the characteristics of emergency medicine (EM) M&Ms nationally, and found significant variation in core structural elements.^8^,^9^,^10^ Given the fear of “blame and shame,” we hypothesized that anonymous case discussion would cultivate a more effective, and less punitive, M&M conference. Secondarily, we were interested in determining whether this core structural element was associated with the overall culture of safety at an institution. We conducted a national survey, evaluating residents’ perceptions of M&M and the culture of safety at their institutions.

## METHODS

### Study Setting and Measurement

Study setting and measurement is discussed in detail in a previous paper.^9^ This is a convenience sample derived from all United States EM residency programs. We invited all programs to participate in a survey of all residents: 33 programs were both willing to participate and able to identify a local champion to serve as a co-investigator to help ensure a high response rate. The survey was conducted in May 2015, using a tool that was previously piloted with residency program directors (PD).^9^ This included questions used in a previous survey of EM PDs^9^ as well as questions from the validated Agency for Health Care Research and Quality (AHRQ) Safety Culture survey.^11^ These questions are designed to assess aspects of a strong safety culture, including a non-punitive environment, comfort submitting and discussing errors, and an environment in which mistakes lead to positive change.

**Population Health Research Capsule**

What do we already know about this issue?

*Structured case review is mandated by the Accreditation Council for Graduate Medical Education, but there are no best practices in the design of these conferences*.

What was the research question?

Does anonymous case discussion cultivate a more effective, and less punitive, morbidity and mortality (M&M) conference?

What was the major finding of the study?

*Anonymous M&Ms are associated with a perception of a less-punitive M&M and with better ratings in several conference-specific outcomes*.

### Analysis

We analyzed data using descriptive statistics and bivariate analyses. Likert scores were summarized using mean and 95% confidence intervals (CI). We calculated composite culture of safety scores by using the average of the four AHRQ safety domains surveyed. Anonymous and non-anonymous comparison was made using paired t-test. Data analysis was performed with STATA MP 13.1 (StataCorp, College Station, TX). We performed qualitative analysis of the free-text comments using conceptual content analysis.

To begin, a set of thematic codes was developed by three of the emergency physician investigators (ELA, JDS, KW) through an iterative reading of all reports. Subsequently, one author (ELA) used content-analysis techniques to code all transcripts in NVivo qualitative data analysis software version 10, 2014 (QSR International Pty Ltd. Doncaster, Australia). Multiple themes could be applied to a single response as appropriate. The final coded text and example quotations were reviewed with two other investigators (KW, JDS) iteratively until there was agreement on the coding structure. Descriptive statistics were generated to summarize the data and quantify the frequency of themes.

## RESULTS

There were 1,248 residents at 33 programs surveyed during the study period. Of the 1002 who replied (80.3% response rate), 231 respondents reported anonymous case presentations, and 744 reported non-anonymous case presentations. There were no differences in the structural elements of the residency training programs between anonymous and non-anonymous respondents (Table 1).

When asked about features of M&M specifically, residents reporting anonymous case presentation reported that M&M was less punitive (Table 2; difference in percent agreeing = 8.21; 95% CI, 11.66–4.77; p<0.05). Regarding case submission, residents reporting anonymous case presentation trended toward being more comfortable submitting cases in which they were *not* involved as compared to residents reporting non-anonymous case presentations (difference in percent agreeing = 6.64; 95% CI, 7.24–14.01; p = 0.08). There was little difference between the groups in their degree of comfort when reporting cases they were involved in (difference in percent agreeing = 0.38; 95% CI, 6.44–7.21; p = 0.91).

Greater than 85% of all residents surveyed agreed that M&M was of value to their education, with no significant difference between the two groups (difference in percent agreeing =1.49; CI, 6.49–3.49; p = 0.55). Those reporting anonymous case presentation did report that case discussion was more focused on system errors (difference in percent agreeing = 6.73; CI, 0.85–12.62; p = 0.03); however, the two groups reported no significant difference in the perception that the discussions were more focused on cognitive errors (difference in percent agreeing = 0.004; CI, 7.22–7.31; p = 0.99]) The majority of residents surveyed agreed that mistakes led to positive change (65.9% of residents reporting anonymous case presentations; 68.6% of residents reporting non-anonymous case presentations).

Table 3 shows the positive composite score for the culture of safety survey stratified by the two different types of case presentation. There remained a significant difference between the two groups related to the perception of the punitive nature of the conference, with residents at programs with anonymous M&Ms significantly less likely to report that the M&M felt punitive.

When asked “Have you had a negative experience with having a case of yours discussed at M&M?,” there was no statistical difference between respondents reporting an anonymous case presentation described negative experiences and respondents reporting non-anonymous case presentations described negative experiences (2.4% vs 0.8%; p = 0.188). Similarly, there was no statistically significant difference between positive experiences reported by respondents reporting anonymous case presentations and respondents reporting non-anonymous case presentations (6.9% vs 8.7%; p = 4.16).

### Narrative Comments

When we systematically analyzed and coded these statements, we found that comments related specifically to anonymity were very common and both positive and negative. The majority of comments from anonymous programs were related to non-punitive responses to error, with residents noting that “*I feel bad about a decision that I made that I should have done differently; however, people are not punitive, they try to keep the discussion academic”;* and “*the environment is generally very constructive and not punitive, which makes it much easier to accept criticisms of the care I provided*.” Another theme identified associated with anonymous case discussions was related to the impact of the providers not being present for the case discussion, noting that as a negative experience: the “*Attending who drove most of decision making of case was not present*.” While these two things, anonymity and the absence of the team involved, are not clearly related it does stand to reason that if the team will not be identified it becomes easier for them not to attend.

Additional themes identified were related to anonymity decreasing the punitive nature of these conferences and increasing the focus on systems and ability to learn from cases: “*M&M for us is completely anonymous and focused on systems errors and ways to avoid a similar error in the future. It does not feel punitive or finger-pointing. It was very interesting to hear the discussion of a case I was involved in, and allowed me to better process a poor patient outcome and give me ideas on how to prevent a similar error in the future*.” However, another resident noted that the same anonymous framework can lead to a loss of the context related to the original medical decision-making: “*I don’t like that our institution does not allow the person involved in the case to OWN the case. Instead you listen to people talking about what they would have done etc. but the person involved in the case is not allowed to stand up and explain their motivations because it has to be anonymous.”*

Other residents provided examples of hybrid approaches to anonymity and described the ability of non-anonymous conferences to provide closure; however, “*at our institution the attendings and residents involved in the case are free to identify themselves and their experience but there is no pressure. When my case was presented, I thought it was helpful to discuss my experience, thought process during the case, and to ask if others in the room would have approached it differently. This gave me a sense of closure and afterwards I felt more resolution regarding the care of that patient.”* Another resident describing a hybrid conference noted the limitations of the non-anonymous structure, impeding honest discussion, perhaps related to self-censorship: “*at our institutions the presenter never identifies the resident or attending on the case but the resident and attending frequently self-identify and start discussing the case. That then makes it very difficult for others to comment honestly and changes the tone*.”

A common theme in the comments from non-anonymous programs was related to residents perceptions of punitive responses to error, such as “*I felt as though I was blamed by one of our senior attendings for this in front of the M&M attendees, even though I had ultimately no power in the decision made by the spine surgery team.”* Alternatively, another theme identified was related to the non-anonymous conference’s ability to cultivate professionalism and accountability. One resident asserted that, “*it is helpful to watch more seasoned providers accept responsibility”;* and another resident noted, “*It teaches patient safety, personal accountability, and management of difficult cases*.”

Another theme that arose exclusively in the non-anonymous group was related to the absence of change resulting from case discussion. One resident noted that “*I have as of yet been informed of any system change to address the issue”* and noting concern that “w*hen a system process is changed as a result of questions and answers, it usually is not effectively communicated to the group (Attendings and Residents) and is often not widely adopted*.” Despite these concerns, residents reporting non-anonymous case presentations did point out the conferences’ ability to provide emotional support to the clinicians involved in the cases discussed, commenting that “ *This gave me a sense of closure and afterwards I felt more resolution regarding the care of that patient*” and “*allowed me to better process a poor patient outcome*.”

## DISCUSSION

In this national survey of residents’ perception of M&M conferences and their institutional cultures of safety, we found that residents reporting anonymous M&Ms were less likely to report that the M&M felt punitive and more likely to report that case discussions were focused on system issues. We found no other association between the AHRQ culture of safety scores and anonymity in M&M. As we think about the core elements of a strong safety culture that could be cultivated through M&M it becomes important that these conferences are designed to encourage robust case reporting, cultivate a nonpunitive environment for discussion, and provide clear follow-up for issues discussed.^12^ Our study suggests that residents at institutions with anonymous M&Ms feel the case discussions are less punitive and that they focus more on systems errors.

In keeping with our hypothesis, we believe that this impact stems from a relationship between the fear of individual blame for case outcomes and being explicitly named in case discussion. It should be noted, however, that it was still a small minority of residents, from either conference structure, who felt these conferences are punitive. Instead, only 16% of residents at anonymous programs and 31% of residents at non-anonymous programs felt that these discussions were punitive. Although other indicators, such as the educational value of the conference, showed no change between the two structures, these also had the clear majority of respondents from both programs (86% anonymous, 88% anonymous) agreeing that the conferences were of value.

Our study showed that, despite the impact of anonymous M&M on some indicators of safety culture, there was no impact on several others. This likely reflects the fact that M&M conferences are only one small determinant of an institution’s culture of safety and this structure alone is not enough to modify the overall culture. This was reinforced by the qualitative analysis, which demonstrated that there were both residents who felt that anonymity cultivated a safety culture, and those who felt it hindered it. This further demonstrates the complexity of safety culture and reinforces that any single input, such as M&M conferences, is only one factor in determining the overall culture.

The analysis of free-text comments provided deeper insight into the nuances surrounding anonymous case presentations, painting pictures of both residents for whom anonymity provided a non-punitive environment that enabled the discussion of systems issues, as well as those for whom anonymity was frustrating and obstructed the ability of the providers to accurately relay the details of the case. The same was true for those reporting non-anonymous case discussions, with some residents recalling situations in which this structure led to them feeling personally attacked or abandoned; however, others described the important impact that this structure had on cultivating personal accountability and professionalism.

## LIMITATIONS

There were several limitations to our study. As with all survey-based research, our study was prone to response bias. Although we had a robust response rate, with a >80% response rate across the study population and > 70% at each institution, only 33 programs out of the 151 programs in the country elected to participate. We suspect that those that did elect to participate were more likely to have stronger safety cultures and that, therefore, our results were biased toward a smaller effect size of anonymity. The survey questions themselves, although taken from a previously piloted survey for PDs and validated AHRQ questions, did not undergo formal psychometric testing as this set of questions. The qualitative analysis of the free-text comments has limitations typical for qualitative analysis, that our findings are hypothesis generating and not generalizable.

## CONCLUSION

In this national survey of EM residents, we found that anonymous M&Ms are associated with a non-punitive perception of the conference. Future study should focus on the impact, within a single program, of anonymous case discussion, as well as other structural elements of M&M conference.

## Figures and Tables

**Table 1:** Demographics and structure of emergency medicine morbidity and mortality conferences.

	Residents reporting anonymous case presentations (%, N)	Residents reporting non-anonymous case presentations (%, N)	X^2^
X^2^			
Total respondents	23.69%, 231	76.31%, 744	
Postgraduate year			X^2^=2.15 P = 0.54
PGY 1	23.13%, 71	76.87%, 236	
PGY 2	25.18%, 71	74.82%, 211	
PGY 3	25.00%, 65	75.00%, 195	
PGY 4	19.05%, 24	80.95%, 102	
Residency program structure			X^2^=0.42 Pr = 0.52
PGY 1–3	24.55%, 123	75.45%, 378	
PGY 1–4	22.78%, 108	77.22%, 366	
Region			X^2^=16.48 Pr = 0.001
Northeast	26.87%, 108	73.13%, 294	
Midwest	22.55%, 53	77.45%, 182	
South	28.57%, 50	71.43%, 125	
West	12.27%, 20	87.73%, 143	
(2) Number of your cases submitted to M&M in the past 12 months			X^2^=2.02 Pr = 0.36
0	25.13%, 146	74.87%, 435	
1	22.77%, 46	77.23%, 156	
≥2	20.21%, 38	79.79%, 150	
(3) Number of your cases submitted to PSRS in the past 12 months			X^2^=0.56 Pr = 0.75
0	24.07%, 181	75.93%, 571	
1	21.37%, 25	78.63%, 92	
≥2	25.51%, 25	74.49%, 73	
(4) Number of your cases presented at M&M during residency			X^2^=2.01 Pr = 0.37
0	24.94%, 106	75.06%, 319	
1	24.72%, 67	75.28%, 204	
≥2	20.58%, 57	79.42%, 220	
(16) Most important objective of M&M			X^2^=9.17 Pr = 0.10
Discuss adverse outcomes	41.04%, 87	34.06%, 233	
Identify systems errors	26.89%, 57	23.10%, 158	
Discuss interesting cases	10.38%, 22	11.26%, 77	
Identify cognitive errors	4.25%, 9	7.31%, 50	
Teach individual professional accountability	9.43%, 20	14.33%, 98	
Other	8.02%, 17	9.94%, 68	

*X^2^*, chi-square test; *PGY*, postgraduate year; *Pr*, probability; *PSRS*, the Patient Safety Reporting System; *M&M*, morbidity and mortality.

**Table 2:** Anonymity and culture of safety domains.

Questions related to culture of safety	Residents reporting anonymous case presentations			Residents reporting non-anonymous case presentations			Difference in % agree [CI]	P-value

	Agree (Lik 4+5) % (n)	Neutral (Lik 3) % (n)	Disagree (Lik 1+2) % (n)	Agree (Lik 4+5) % (n)	Neutral (Lik 3) % (n)	Disagree (Lik 1+2) % (n)		
(7) M&M feels punitive (primary outcome)	3.93% (9)	11.79% (27)	84.28% (193)	12.15% (90)	18.76% (139)	69.10% (512)	−8.21% [−11.66; −4.77]	<0.05
(5) Comfort submitting cases I was not involved in	48.70% (112)	23.48% (54)	27.83% (64)	42.05% (312)	28.44% (211)	29.51% (219)	6.64% [−7.24; 14.01]	0.08
(6) Comfort submitting cases I was involved in	69.43% (159)	21.40% (49)	9.17% (21)	69.04% (513)	20.73% (154)	10.23% (76)	0.38% [−6.44; 7.21]	0.91
(8) Case discussion is focused on cognitive errors	59.13% (136)	29.57% (68)	11.30% (26)	59.08% (439)	26.24% (195)	14.67% (109)	0.04% [−7.22; 7.31]	0.99
(9) Case discussions are focused on systems errors	81.74% (188)	14.78% (34)	3.48% (8)	75.00% (558)	17.61% (131)	7.39% (55)	6.73% [0.85; 12.62]	0.03
(10) Mistakes have led to positive changes	65.94% (151)	29.26% (67)	4.80% (11)	68.64% (510)	26.51% (197)	4.85% (36)	−2.7% [−9.68; 4.28]	0.44
(12) M&M is a valuable educational didactic session	86.52% (199)	10.00% (23)	3.48% (8)	88.02% (654)	9.15% (68)	2.83% (21)	−1.49% [−6.49; 3.49]	0.55

*Lik*, Likert; *CI*, confidence interval; *M&M*, morbidity and mortality.

**Table 3:** Primary and secondary outcomes.

Questions related to Culture of Safety	Residents reporting anonymous case presentations	Residents reporting non-anonymous case presentations	Difference in % Agree [CI]	P value

	Average Likert score	Average Likert score		
(7) M&M feels punitive (primary outcome)	1.66	2.05	−0.39 [−0.55; −0.23]	<0.05
(5) Comfort submitting cases I was not involved in	3.39	3.25	0.15 [−0.04; 0.33]	0.12
(6) Comfort submitting cases I was involved in	3.92	3.88	0.04 [−0.11; 0.19]	0.61
(8) Case discussion is focused on cognitive errors	3.60	3.57	0.03 [−.10; 0.17]	0.63
(9) Case discussions are focused on systems errors	4.05	3.89	0.16 [0.03; 0.28]	0.01
(10) Mistakes have led to positive changes	3.82	3.84	−0.01 [−0.13; 0.11]	0.84
(12) M&M is a valuable educational didactic session	4.38	4.36	0.02 [−0.10; .14]	0.71

*CI*, confidence interval; *M&M*, morbidity and mortality.
